# PReS-FINAL-2278: Description of a Colombian cohort of patients with childhood systemic lupus erytematosus

**DOI:** 10.1186/1546-0096-11-S2-P268

**Published:** 2013-12-05

**Authors:** AS Diaz Maldonado, A Monje Gaitan, F Gonzalez, N Gamba

**Affiliations:** 1 Reumatologia Pediatrica, Hospital de La Misericordia, Bogota, Colombia

## Introduction

Systemic lupus erythematosus (SLE) is a multisystem disease of autoimmune etiology, which carries a high morbidity and mortality.

## Objectives

To describe clinical and immunoserological features at the time of diagnosis within a cohort of pediatric patients attending the service of rheumatology of a Colombian pediatric hospital.

## Methods

Cross-sectional study. 89 patients with diagnosis SLE (1986 ACR criteria) from a rheumatology center at a pediatric hospital were evaluated. Medical records were reviewed registering the following variables: sex, mean age, score SLEDAI, organ involvement, current pharmacological treatment and autoimmune profile. Descriptive analysis was done for qualitative and categorical variables (percentages and averages) using STATA11.

## Results

76 patients (85.4%) were female and 13 (14.6%) were men. Mean age 11.3 y/o (min 2 max 16). Median score SLEDAI at onset was 23 (min 4 max 61). Organ involvement: Renal 75 patients (84,3%), hematologic 75 (84,3%), skin 60 (67,4%), neurolupus 26 (29,9%), serositis 17 (19,1%), arthritis 50 (56,18%), Raynaud 8 (10,1%), photosensitivity 34 (52,3%). Current pharmacological treatment: antimalarials 80 (93%), Azathioprine 57 (64%), Cyclophosphamide 33 (38,8%), Mycophenolate Mofetil 8 (9,6%), Rituximab 4 (4,5%), Cyclosporine 1 (1,1%). Autoimune profile: Antinuclear antibodies (ANAs) reactivity 74 (88,1%), Anti-DNA antibodies 56 (66,7%), Antiphospholipid antibodies (aPL) 25 (38,1%).

## Conclusion

The demographic characteristics and laboratory tests of this cohort are according to previously reported in worldwide and latin American literature. The score SLEDAI at the diagnosis was found in median activity. The most common medications prescribed were antimalarials followed by azathioprine and cyclophosphamide.

## Disclosure of interest

None declared.

